# Long-term outcomes of concomitant chemoradiotherapy with temozolomide for newly diagnosed glioblastoma patients: A single-center analysis

**DOI:** 10.1097/MD.0000000000007422

**Published:** 2017-07-07

**Authors:** Tae Hoon Roh, Hun Ho Park, Seok-Gu Kang, Ju Hyung Moon, Eui Hyun Kim, Chang-Ki Hong, Sung Soo Ahn, Hye Jin Choi, Jaeho Cho, Se Hoon Kim, Seung Koo Lee, Dong Seok Kim, Sun Ho Kim, Chang-Ok Suh, Kyu Sung Lee, Jong Hee Chang

**Affiliations:** a Yonsei University Graduate School, Seoul; b Department of Neurosurgery, Ajou University Hospital, Ajou University School of Medicine, Suwon; c Department of Neurosurgery; d Department of Radiology; e Department of Medical Oncology; f Department of Radiation Oncology; g Department of Pathology, Yonsei University College of Medicine; h Brain Tumor Center, Severance Hospital, Yonsei University Health System; i Brain Research Institute, Yonsei University College of Medicine, Seoul, Republic of Korea.

**Keywords:** chemoradiotherapy, DNA methylation, glioblastoma, survival analysis, temozolomide

## Abstract

The present study analyzed outcomes of surgery followed by concomitant chemoradiotherapy (CCRT) with temozolomide (TMZ) in patients with newly diagnosed glioblastoma (GBM) at a single institution. Outcomes were retrospectively reviewed in 252 consecutive patients with newly diagnosed GBM who underwent surgery followed by CCRT with TMZ at the authors’ institution between 2005 and 2013. At initial operation, 126 (50.0%), 55 (21.8%), 45 (17.9%), and 26 (10.3%) patients underwent gross total resection (GTR), subtotal resection, partial resection (PR), and biopsy, respectively. Their median overall survival (OS) was 20.8 months (95% confidence interval [CI] 17.7–23.9 months) and their median progression-free survival was 12.7 months (95% CI 11.2–14.2 months). The O^6^-methylguanine-DNA methyltransferase (MGMT) promoter was methylated in 78 (34.1%) of the 229 patients assayed, and an isocitrate dehydrogenase 1 mutation was detected in 7 (6.6%) of the 106 patients analyzed. Univariate analyses showed that patient age, involvement of eloquent areas, involvement of the subventricular zone, presence of leptomeningeal seeding, Karnofsky Performance Status, extent of resection (EOR), MGMT promoter methylation, and presence of an oligodendroglioma component were prognostic of OS. Multivariate analysis showed that age, involvement of eloquent areas, presence of leptomeningeal seeding, EOR, and MGMT promoter methylation were significantly predictive of survival. OS in patients with GBM who undergo surgery followed by CCRT with TMZ is enhanced by complete resection. Other factors significantly prognostic of OS include that age, involvement of eloquent areas, presence of leptomeningeal seeding, and MGMT promoter methylation.

## 1 Introduction

Glioblastoma (GBM) is the most malignant and common primary brain tumor in adults. Standard therapy of patients with newly diagnosed GBM includes resection followed by concomitant chemoradiotherapy (CCRT) and adjuvant temozolomide (TMZ).^[1]^ Despite treatment, however, the median overall survival (OS) of patients with GBM is only 14.6 to 21.1 months.^[2,3]^

Characteristics prognostic of survival in patients with GBM include clinical factors such as age, performance score, and extent of resection (EOR), along with factors associated with tumor location, such as invasion of eloquent areas or the subventricular zone (SVZ) and coexistence of leptomeningeal seeding.^[4–6]^ Survival rate is strongly dependent on treatment modality; adjuvant chemoradiotherapy with TMZ is considered standard because it significantly increases patient survival rate. Furthermore, advances in surgical techniques and technology have been reported to improve survival.^[7,8]^

Molecular prognostic factors in GBM include mutations in the genes encoding isocitrate dehydrogenase (IDH) 1/2 and methylation of the O^6^-methylguanine-DNA methyltransferase (MGMT) promoter.^[9,10]^ MGMT promoter methylation has also been found to predict response to TMZ-based chemotherapy.^[9]^ A meta-analysis published in 2014, which investigated the prognostic value of MGMT promoter methylation in different races, reported that, in Asians, MGMT promoter methylation was not related to OS by univariate analysis or to progression-free survival (PFS) by multivariate analysis.^[11]^ However, this meta-analysis included only 6 studies in Asians, compared with 46 in Caucasians, as only a few studies have reported molecular information on GBM in Asian patients.^[12]^

To assess the survival benefits of standard treatment, this study analyzed 252 consecutive GBM patients who underwent surgery followed by standard chemoradiotherapy (CCRT) plus TMZ at a single institution. The associations of MGMT promoter methylation and IDH1 mutation status with survival were also investigated.

## 2 Materials and methods

### 2.1 Patient recruitment

The present retrospective study was approved by our institutional review board, which waived the requirement for patient informed consent due to the retrospective nature of this study (2015-2474-001). All patients coded as having GBM histology at Yonsei University Severance Hospital from 2005 to 2013 were screened. Subjects were included if they had been histologically diagnosed with GBM according to the World Health Organization (WHO) classification of central nervous system tumors,^[13]^ as confirmed by 2 pathologists (SHK and JC). Patients having GBM with oligodendroglioma component (GBMO) and giant cell GBM (GCGBM) were also included. Patients were excluded if preoperative or follow-up images were unavailable, if they were aged <18 years, if they had coexisting malignancy or gliosarcoma. Patients and their guardians were informed of treatment options before surgery and when pathological diagnosis was obtained after surgery. The patients who agreed to start standard CCRT with TMZ were included in the analysis. All patients were followed up until death or the time of analysis (August 2016).

### 2.2 Treatment protocols

Tumors were removed following the protocol of maximal safe resection, except for patients who underwent biopsy. Most patients underwent navigation-guided surgery, using intraoperative magnetic resonance imaging (MRI) in 39 patients and 5-aminolevulinic acid in 74. Diffusion tensor image tractography was used for navigation in patients with tumors located adjacent to areas of motor, vision, and language functions to minimize possible damage. Awake craniotomy was performed in 24 patients. Most patients (87%) started CCRT with using Stupp regimen (daily 75 mg/BSA of TMZ plus 60 Gy of radiotherapy fractionated 2 Gy/d) within 3 weeks of surgery.^[1]^ Twenty-eight days following the completion of CCRT, TMZ was started; 6 cycles were administered unless adverse event or progression was detected.

In cases of recurrence, reoperation was considered a priority, when the tumor was resectable. Maximal safe resection was attempted using the method used for initial surgery. Repeat radiotherapy was again recommended after surgery. If the recurrent tumor was considered unresectable, due to deep location, involvement of eloquent areas, or small size, gamma-knife radiosurgery was recommended. Adjuvant TMZ administration was also considered following repeat radiotherapy. If the tumor rerecurred, bevacizumab was considered as salvage treatment.

### 2.3 Image analysis

All patients underwent pre- and postoperative MRI, with images stored in a picture archiving and communication system. Images were assessed and reviewed by 2 experienced radiologists. Parameters evaluated preoperatively included tumor size (maximal diameter of enhancing lesion), primary lesion location, relationship to eloquent areas, SVZ involvement, and leptomeningeal seeding. The size of the tumor was classified using cutoff value of 3.4 cm, chosen by Contal and O’Quigley method.^[14]^

The EOR was determined by early (<48 hours) postoperative MRI. Gross total resection (GTR) was defined as the absence of a residual lesion, based on T1-weighted contrast enhancement images. When the tumor was left in the surgical field, the tumor was considered to have undergone a STR even if no tumor was seen in the postoperative MRI findings. Subtotal resection (STR) was defined as the presence of a residual tumor but EOR was >90%. Partial resection (PR) was defined as the presence of >10% of tumors (EOR <90%). In the case of a discrepancy, 3 observers (THR, MCO, and JHC) simultaneously reviewed the images to achieve consensus.

Follow-up MRIs were performed before the start of the first and fourth TMZ cycles and 1 month after the end of the sixth cycle. Patients underwent follow-up MRI every 3 months for the first 2 years, and every 6 or 12 months thereafter. Patients suspected of disease progression, as defined by RANO criteria, underwent immediate MRI.^[15]^

A transient progressive lesion within 3 months after radiotherapy was considered pseudoprogression. Patients with an apparent newly appearing enhancing mass were assessed by MR spectroscopy, ^11^C-methionine positron emission tomography, or perfusion MRI to differentiate between radiation necrosis and recurrence.

### 2.4 Molecular analysis

Genomic DNA was isolated from paraffin-embedded samples of 229 patients. The DNA methylation status of the CpG islands at the MGMT promoter was determined by methylation-specific polymerase chain reaction, as described, with some modifications.^[16,17]^

Representative tissue sections were assessed by immunohistochemistry, using a Ventana BenchMark XT autostainer (Ventana Medical System, Inc. Tucson, AZ) according to the manufacturer's protocols. Primary antibodies included anti-human IDH1 R132H mouse monoclonal (clone H09L, Dianova, 1:80 dilution) and anti-Ki-67 (clone Mib-1, Dako, 1:150 dilution). Samples showing cytoplasmic expression of IDH1 R132H in glioma cells were classified as positive for mutation, with all others classified as “wild-type.” MIB-1 (Ki67) score was defined as the percentage of positive nuclei among 1000 tumor cells, or as many as possible in the case of small specimens.

### 2.5 Statistical analysis

Survival outcomes were analyzed by the Kaplan–Meier method and compared by the log-rank test. Multivariate Cox proportional hazards models were created to test for associations of factors with PFS and OS. In these models, EOR was dichotomized as complete (gross total) versus incomplete (subtotal, partial, and biopsy). All statistical tests were 2-sided, and the threshold for statistical significance was *P* < .05. All analyses were performed with SPSS for Windows version 20.0 (SPSS Inc., Chicago, IL) and SAS version 9.2 (SAS Institute Inc., Cary, NC).

## 3 Results

### 3.1 Patient characteristics and overall survival

Between January 2005 to December 2013, 313 patients newly diagnosed with pathologically proven GBM were treated at Yonsei University Severance Hospital. Sixty-one patients were excluded, including 11 aged <18 years, 21 who were lost to follow-up, 4 who had a coexisting malignancy, 4 who died of pneumonia before CCRT, 7 who refused CCRT, 13 who received only radiotherapy, and 1 who received TMZ alone. Finally, 252 patients were included in the analysis.

The characteristics of the included patients are shown in Table 1. The median time from diagnosis to the start of CCRT was 20 days 4 to 6 (range, 8–47 days). The median therapeutic dose of radiation was 60 Gy (range, 7.2–84 Gy). The median follow-up period was 20.8 months, and 212 (84.1%) patients died during the study period. The median OS was 20.8 months (95% confidence interval [CI]: 17.7–23.9 months), and the median PFS was 12.7 months (95% CI: 11.2–14.2 months). The actuarial 1-, 3-, and 5-year OS rates were 79.8%, 28.2%, and 16.2%, respectively, and the actuarial 1-, 3-, and 5-year PFS rates were 54.1%, 14.4%, and 6.1%, respectively.

**Table 1:**
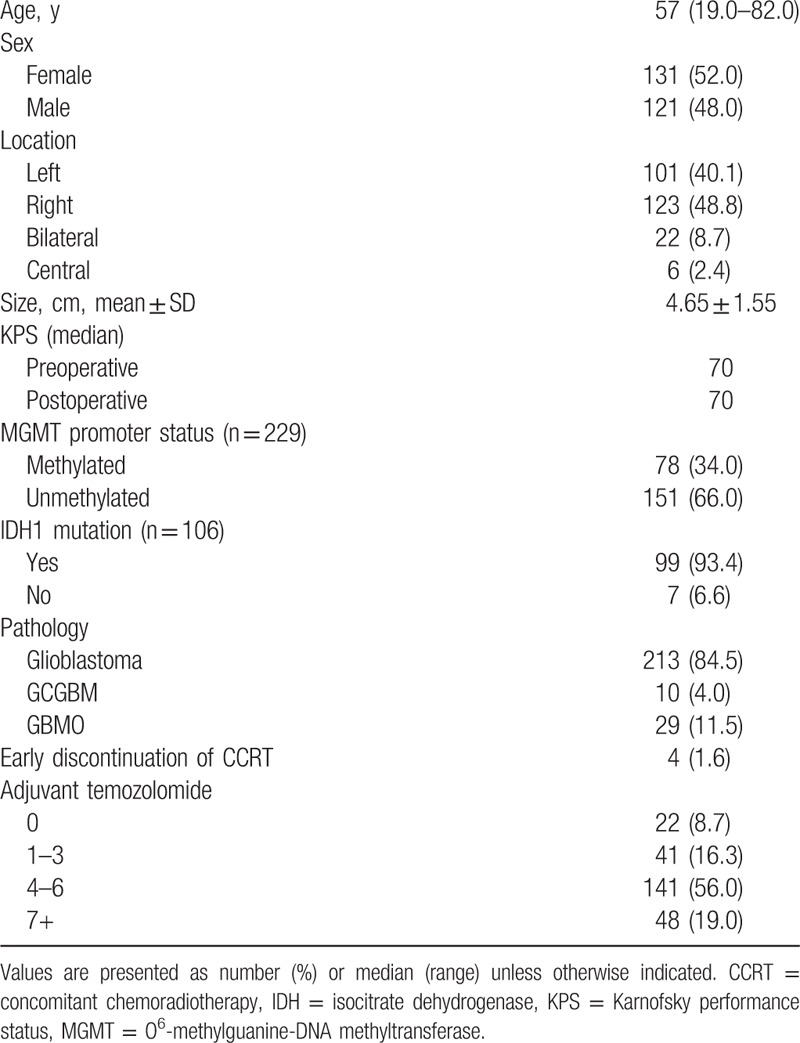
Patient characteristics.

### 3.2 Clinical factors and prognostic relevance

The prognostic relevance of the clinical parameters is summarized in Table 2. As predicted, patients aged <50 years showed better prognosis than those aged ≥50 years. OS was significantly shorter in patients having lesions located in eloquent areas or with SVZ involvement, subependymal enhancement, or leptomeningeal dissemination. Using 3.4 cm as the cutoff-value, tumor size was unrelated to either OS and PFS. A Karnofsky performance status (KPS) score ≥80 was associated with improved OS, but not PFS.

**Table 2:**
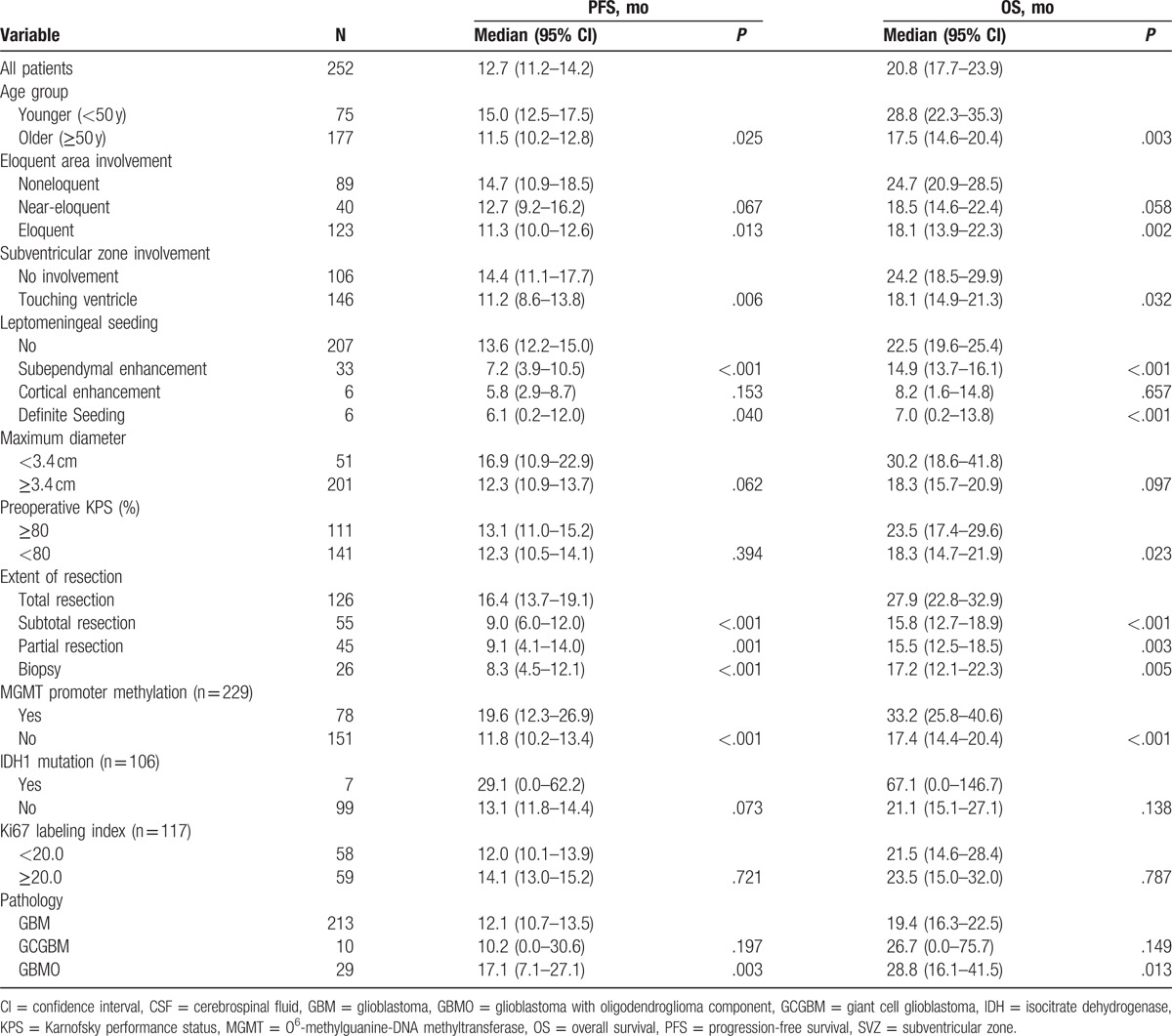
Univariate analyses of factors prognostic for OS and PFS.

**Figure 1:**
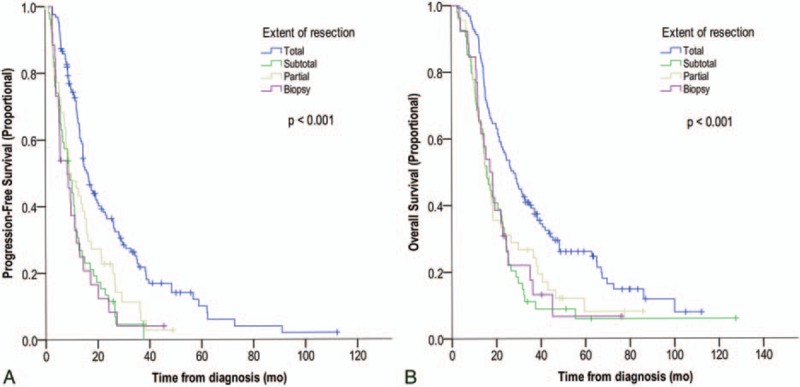
Kaplan–Meier analysis of the effects of extent of resection on progression-free survival (PFS) (A) and overall survival (B). Total resection significantly benefited PFS and overall survival when compared with subtotal resection, partial resection, and biopsy (*P* < .001 each).

At initial operation, 126 (50.0%), 55 (21.8%), 45 (17.9%), and 26 (10.3%) patients underwent GTR, STR, PR, and biopsy, respectively. Both OS and PFS were higher in subjects who underwent GTR than in those who underwent STR, PR, and biopsy. Median OS was 27.9 months (95% CI, 22.8–32.9 months) in patients who underwent GTR, but there were no differences in OS or PFS among patients who underwent STR, PR, and biopsy (Fig. 1). Patients who underwent GTR had 1-, 3-, and 5-year OS rates of 91.3%, 38.6%, and 25.3%, respectively, whereas patients who underwent incomplete resection (STR, PR, and biopsy) had 1-, 3-, and 5-year OS rates of 68.0%, 17.6%, and 7.2%, respectively.

MGMT promoter methylation was detected in 78/229 (34.1%) patients and was associated with superior PFS and OS. MGMT status was not determined in 23 patients due to insufficient amount of tissue. Most of these patients (18/23) were who had undergone biopsy alone. IDH1 mutations were found in 7/106 (6.6%) patients. The median OS for patients with mutated and wild-type IDH1 were 67.1 and 21.1 months, respectively (*P* = .138). Ki67 labeling index was not associated with PFS or OS. Patients with GCGBMs did not differ from those with GBM in either PFS or OS, whereas patients with GBMO showed significantly longer PFS and OS than other patients.

### 3.3 Multivariate analysis for outcome

Univariate analyses showed that factors significantly prognostic of OS and PFS included patient age, KPS, EOR, MGMT promoter methylation status, eloquent area involvement, SVZ involvement, and leptomeningeal seeding. In performing multivariate logistic regression analysis, patients with missing values were excluded; therefore, multivariate analysis included only the 229 patients with known MGMT promoter methylation status. Young age (<50 years), GTR, subependymal enhancement on preoperative MRI, and MGMT promoter methylation were found to be independently prognostic factors for both PFS and OS (Table 3), whereas eloquent area involvement and leptomeningeal dissemination were significantly prognostic only for OS.

**Table 3:**
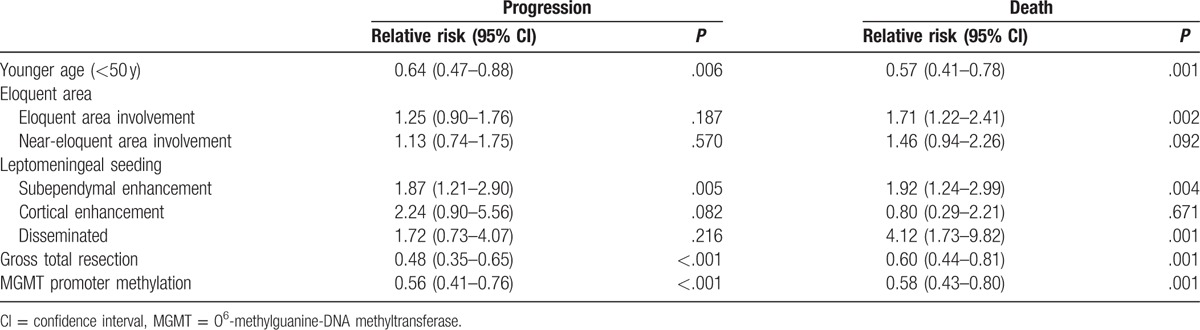
Multivariate analyses of factors prognostic for OS and PFS.

### 3.4 Overall survival related to 3 significant prognostic factors

Treatment outcome was analyzed in 8 groups of patients stratified by the 3 most significant prognostic factors; age, MGMT promoter methylation, and EOR (Table 4). The median OS of the 13 patients with methylated MGMT, young age (<50 years), and GTR was 67.1 months (range, 12.7–121.5 months). In contrast, the median OS of the 45 patients with an unmethylated MGMT gene, older age (≥50 years), and less than STR was 14.8 months.

**Table 4:**
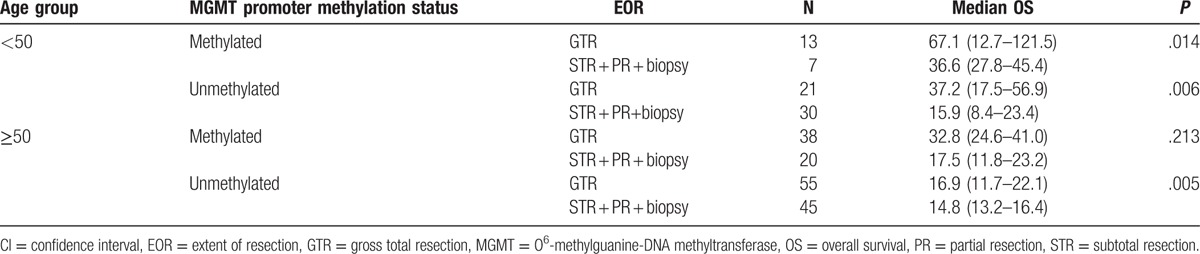
Relationships among 3 factors significant prognostic of OS.

## 4 Discussion

Clinical factors considered significantly prognostic for survival in patients with GBM include age, performance status, EOR, tumor location, degree of necrosis, and enhancement on preoperative MRI.^[6]^ Because the treatment modality influences the survival rate, we analyzed only those patients who agreed to start standard therapy (CCRT plus TMZ). The median OS of GBM patients who underwent TMZ-based CCRT reported in large prospective studies is shown in Table 5.

**Table 5:**
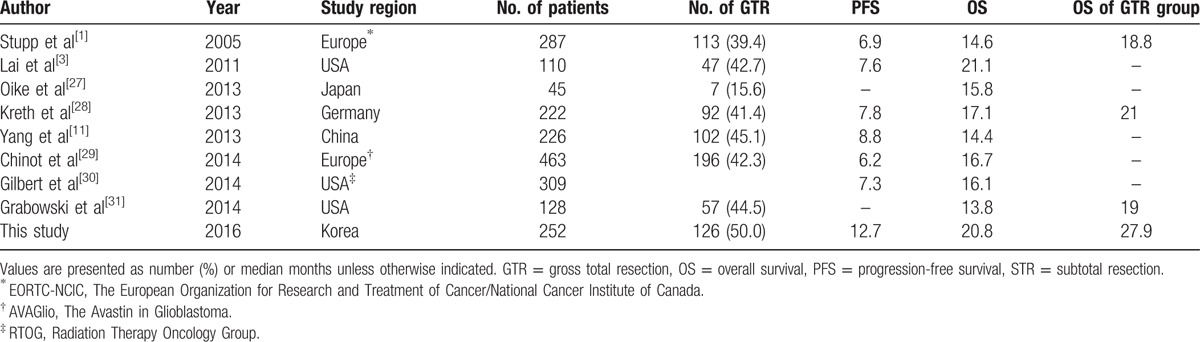
Literature findings of patient characteristics, PFS, OS, and EOR in patients with GBM.

The median OS and PFS of all patients in this study were 20.8 and 12.7 months, much higher than the 14.6 and 6.9 months reported in the EORTC/NCIC study.^[1]^ One plausible cause for this discrepancy may have been the higher percentage of patients in our study who underwent total resection. In our study, 50% of subjects underwent GTR, with a median OS of 27.9 months, significantly higher than the median OS of the patients who underwent STR, PR, and biopsy. In the EORTC/NCIC study, approximately 40% of patients underwent GTR; however, that report provided limited information regarding the method used to select patients for GTR. In our study, the criteria for GTR were thoroughly assessed through intraoperative findings and postoperative MRI within 48 hours. In addition, we performed so-called supratotal resection when tumors were confined in noneloquent areas, removing as much of the brain as possible from adjacent tumors unless they affect function.^[18]^ A future study will assess the survival benefits of supratotal resection.

Salvage treatment after recurrence could also have contributed to longer OS. Of the 217 patients with recurrent GBM, 44 underwent a second operation, 4 underwent a third operation, and 1 underwent a fourth operation for recurrent lesions. In addition, 21 patients underwent repeat radiation treatment or gamma knife surgery for recurrent lesions, and 48 were administered more than 6 cycles of adjuvant TMZ, with the latter treated with a median of 11 cycles (range, 7–24 cycles). Eight patients were treated with bevacizumab plus irinotecan for recurrent lesions. Studies are needed to analyze the effects of salvage treatments in patients with recurrent GBM.

Our results also confirmed that EOR is one of the most significant independent prognostic factors for both PFS and OS.^[5,19–22]^ This factor is important, as it is the only prognostic factor adjustable by surgeons.

Several studies have reported that patients with GBMO have a better prognosis than those with GBM, and that GBMO is associated with a 1p/19q co-deletion.^[23]^ In contrast, other studies have reported no correlation between GBMO and 1p/19q co-deletion or survival.^[24]^ We observed differences in PFS and OS between patients with GBMO and those with GBM. The updated WHO classification in 2016 eliminated the category of GBMO, with these tumors now classified as GBM or anaplastic oligodendroglioma according to the IDH mutation and 1p/19q co-deletion. However, the patients in the present study were diagnosed before the WHO classification was updated, and 15% of patients the EORTC_26981/NCIC_CE.3 trial had GBMO.^[24]^

IDH mutation is an important genetic marker in gliomas. IDH-mutant GBMs are known to show much better prognosis than IDH-wildtype GBMs.^[10]^ According to the WHO classification of tumors of the central nervous system published in 2016, GBM with IDH mutation is classified as a different entity from GBM with IDH-wildtype.^[25]^ In our study, IDH mutation was examined in 42% of patients who performed immunohistochemical staining for IDH1 R132H. It is rare that adult GBM has mutations other than IDH1 R132H.^[26]^ Even if some of the untested younger patients have IDH mutations, there is little chance of causing a significant change in the median survival. Among the examined patients, 6.6% had IDH mutation on the immunohistochemistry, similar to what is known in other studies. In addition, the other studies about GBM before WHO 2016 classification also included IDH-mutant GBM in survival analysis.

We also confirmed that MGMT promoter methylation is an independent prognostic factor of survival in patients with GBM.^[9,12]^ Both PFS and OS were significantly higher in patients with MGMT methylation compared to those without such methylation. TMZ has been shown to enhance survival benefits in patients receiving CCRT, a finding supported in our study, in that most completed CCRT when TMZ was also administered.

The present study had several limitations, including its retrospective design, which may introduce selection bias. Four patients with newly diagnosed GBM were not eligible to start CCRT due to pneumonia. Thirteen patients, most of older age, received radiotherapy alone without TMZ. These patients were excluded from the analysis. However, the characteristics of patients in this study, including median age, age distribution, and performance status, were similar to those of patients in previous studies, such as the EORTC/NCIC trial, who received CCRT. Therefore, this limitation was not considered to have affected our comparison of OS.

## 5 Conclusion

The survival rate in patients with GBM is improved with surgery followed by CCRT plus TMZ. We also confirmed that age, MGMT promoter methylation status, tumor extent, and EOR are significant prognostic factors for survival in GBM patients. Further, we found that EOR was the only modifiable prognostic factor, suggesting that maximum safe resection can improve overall outcome in patients with GBM.
