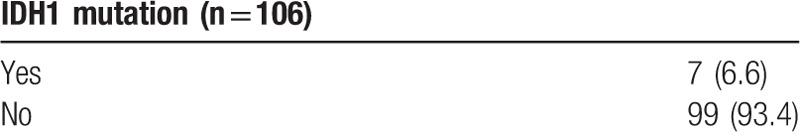# Long-term outcomes of concomitant chemoradiotherapy with temozolomide for newly diagnosed glioblastoma patients: A single-center analysis: Erratum

**DOI:** 10.1097/MD.0000000000007724

**Published:** 2017-07-28

In the article, “Long-term outcomes of concomitant chemoradiotherapy with temozolomide for newly diagnosed glioblastoma patients: A single-center analysis”,^[1]^ which appeared in Volume 96, Issue 27 of *Medicine*, the numbers under the heading “IDH1 mutation (n = 106)” in Table 1 were reversed. That section of the table should have appeared as below.

**Figure:**